# Missed opportunities around school support of FBT for adolescents with anorexia nervosa: facilitation and barriers to supervised eating

**DOI:** 10.1186/2050-2974-1-S1-O62

**Published:** 2013-11-14

**Authors:** Evelyn Bowtell, Julie Green, Rosalie Aroni, Susan Sawyer

**Affiliations:** 1 Centre for Adolescent Health, Royal Children's Hospital, Australia; 2 Department of Paediatrics, The University of Melbourne, Australia; 3 Murdoch Childrens Research Institiute, Australia; 4 Royal Children's Hospital Education Institute, Australia; 5 Parenting Research Centre, East Melbourne, Australia; 6 School of Public Health and Preventitive Medicine Faculty of Medicine, Nursing and Health Sciences, Monash University, Australia

## 

The aim of this qualitative study was to explore the interface between the health and education sectors to better understand how to support adolescents with chronic health conditions. In-depth interviews were conducted with parents (n=38), school (n=16) and health staff (n=11). Parents of adolescents with three conditions (anorexia nervosa [AN, n=11], cancer [11] and cystic fibrosis [CF, n =16]); were interviewed as these conditions each benefit from specific health supports to maintain schooling which is central to peer relations, emotional wellbeing and future financial independence. Parents of adolescents with AN reported that support for parent supervised eating at school during phase one of Family-Based Treatment was either absent or inconsistent. Parents perceived lack of physical space, poor understanding by school staff, and their child's concerns about privacy were barriers to supervised eating. While teachers recognized the reasons for health support for students with cancer, teacher supervised eating was viewed as a medical intervention for which they lacked training. Parents of adolescents with CF were able advocates for their child, while the lack of support for supervised eating for the AN cohort appeared to compromise educational participation. Parents perceived such support would help maintain their children's connectedness to peers and school.

This abstract was presented in the **Children and Youth Treatment and Service Development** stream of the 2013 ANZAED Conference.

